# Are we equally at risk of changing smoking behavior during a public health crisis? Impact of educational level on smoking from the TEMPO cohort

**DOI:** 10.1186/s12889-023-15799-1

**Published:** 2023-05-30

**Authors:** Astrid Juhl Andersen, Irwin Hecker, Solène Wallez, Anke Witteveen, Antonio Lora, Ellenor Mittendorfer-Rutz, Giovanni Corrao, Henrik Walter, Josep Maria Haro, Marit Sijbrandij, Matteo Monzio Compagnoni, Mireia Felez-Nobrega, Raffael Kalisch, Richard Bryant, Maria Melchior, Murielle Mary-Krause

**Affiliations:** 1 Sorbonne Université, INSERM, Institut Pierre Louis d’Épidémiologie et de Santé Publique, IPLESP, Equipe de Recherche en Epidémiologie Sociale, ERES, F75012 Paris, France; 2 Department of Clinical, Neuro-, and Developmental Psychology and WHO Collaborating Center for Research and Dissemination of Psychological Interventions, Vrije Universiteit, Amsterdam, the Netherlands; 3 Direttore Dipartimento di Salute Mentale e Dipendenze, Azienda Socio-Sanitaria Territoriale (ASST) di Lecco, Via dell’Eremo 9/11, 23900 Lecco, Spain; 4 Division of Insurance Medicine, Department of Clinical Neuroscience, Karolinska Institutet, Stockholm, Sweden; 5 Unit of Biostatistics, Epidemiology and Public Health, Department of Statistics and Quantitative Methods, National Centre for Healthcare Research and Pharmacoepidemiology, University of Milano-Bicocca, University of Milano-Bicocca, Milan, Italy; 6 Leibniz Institute for Resilience Research (LIR), Mainz, Germany; 7 Department of Psychiatry and Psychotherapy, Charité-Universitätsmedizin Berlin, corporate member of Freie Universität Berlin and Humboldt-Universität zu Berlin, Charitéplatz 1, D-10117 Berlin, Germany; 8 Parc Sanitari Sant Joan de Déu, Research and Innovation Unit, CIBERSAM, University of Barcelona, Sant Boi de Llobregat, Barcelona, Spain; 9 School of Psychology, University of New South Wales, Sydney, 2052 NSW Australia; 10 Neuroimaging Center (NIC), Focus Program Translational Neuroscience (FTN), Johannes Gutenberg University Medical Center, Mainz, Germany; 11 Sorbonne Université – Faculté de Médecine, Site Saint-Antoine, UMR-S 1136 – N° BC 2908, 27 rue Chaligny, Paris, 75012 France

**Keywords:** Tobacco, COVID-19, Smoking behavior, France, Social inequalities

## Abstract

**Background:**

The COVID-19 pandemic as a public health crisis has led to a significant increase in mental health difficulties. Smoking is strongly associated with mental health conditions, which is why the pandemic might have influenced the otherwise decline in smoking rates. Persons belonging to socioeconomically disadvantaged groups may be particularly affected, both because the pandemic has exacerbated existing social inequalities and because this group was more likely to smoke before the pandemic. We examined smoking prevalence in a French cohort study, focusing on differences between educational attainment. In addition, we examined the association between interpersonal changes in tobacco consumption and educational level from 2018 to 2021.

**Methods:**

Using four assessments of smoking status available from 2009 to 2021, we estimated smoking prevalence over time, stratified by highest educational level in the TEMPO cohort and the difference was tested using chi^2^ test. We studied the association between interpersonal change in smoking status between 2018 and 2021 and educational attainment among 148 smokers, using multinomial logistic regression.

**Results:**

Smoking prevalence was higher among those with low education. The difference between the two groups increased from 2020 to 2021 (4.8–9.4%, p < 0.001). Smokers with high educational level were more likely to decrease their tobacco consumption from 2018 to 2021 compared to low educated smokers (aOR = 2.72 [1.26;5.89]).

**Conclusion:**

Current findings showed a widening of the social inequality gap in relation to smoking rates, underscoring the increased vulnerability of persons with low educational level to smoking and the likely inadequate focus on social inequalities in relation to tobacco control policies during the pandemic.

**Supplementary Information:**

The online version contains supplementary material available at 10.1186/s12889-023-15799-1.

## Introduction

Since 2020, the world has been greatly affected by the global health crisis related to the COVID-19 pandemic. To fight the pandemic, the French government introduced nationwide lockdowns several times from March 2020 onwards. During this period, severe restrictions on social contact, movement, work ability, and access to services and healthcare, particularly preventive and general medicine, were put in place [1, 2], and increased levels of anxiety and depression have been reported both in France and globally [3–5]. Anxiety and depression are found to be associated with increased substance use, including both tobacco smoking uptake and frequency [6, 7], explained by a self-medication model, suggesting that individuals smoke to alleviate psychiatric symptoms [8]. The increased levels of anxiety and depression reported during the pandemic may have affected the otherwise decline in smoking prevalence observed in recent years [9]. Furthermore, fear of infection has been cited as a stressor affecting mental health [10], and perceived risk of complications from COVID-19 related to smoking itself resulted in higher smoking abstinence [11]. Nevertheless, it has been posited that individuals may have increased their smoking behavior as a coping mechanism for psychological distress [12] as well as a consequence of boredom and monotony of lockdown [13]. Concomitantly smoking behavior might have been influenced by environmental factors such as greater availability of smoking opportunities associated with remote work, leading to smoke more freely inside homes, while avoiding the prohibition of smoking indoors at the workplace. In addition, individuals with shorter education were overrepresented in essential jobs during the pandemic that required them to work face-to-face settings [14], which could counteract or even cancel the widening of smoking inequalities during the pandemic. Conversely, some individuals may have decreased smoking behavior as a result of reduced access to tobacco retailers, even if they remained open throughout the lockdown, and fewer social gatherings [15]. It has been found that the length of financial strain during the pandemic was associated with higher change of decreasing smoking intensity [16]. Furthermore, individuals with low socio-economic status were disproportionately impacted by job losses or partial employment during the pandemic, resulting in the loss of some of their income sources due to unstable or supplementary employment [17]. Given the proposed link between smoking and the risk of chronic diseases, including those that serve as risk factors for greater COVID-19 infection severity, it is possible that the pandemic has prompted increased smoking cessation attemps. A recently published systematic review and meta-analysis examining existing literature on tobacco smoking changes during the first pre-vaccination phases of the COVID-19 pandemic reported highly mixed results [12], including a relative reduction in overall smoking prevalence during the pandemic [12]. Previous studies on smoking in the context of the COVID-19 pandemic have mainly focused on changes in the average number of cigarettes smoked, with some identifying a general increase [18–20], while others observed an increase among some groups and a decrease among others [21–25]. These mixed results may reflect a complex interaction between individual characteristics and structural factors [12], which emphazises the need for subgroups analysis, which may explain the divergent results.

Both mental health and tobacco use have been found to associate with lower educational level. Having lower educational attainment or lower income were found to be risk factors for higher levels of anxiety and depression at the start of lockdown [26], which has been suggested to be associated with increased experiences of hardships such as job losses, reductions in household income, and financial struggles. The increase in mental health difficulties among members of disadvantaged groups linked to the pandemic may have negatively impacted associated smoking patterns [27, 28]. A study based on 10 nationally-representative repeated cross-sectional surveys conducted in France [9] found that people with lower socio-economic position (SEP) showed increased smoking prevalence in 2020 when compared to previous years, while in other groups smoking prevalence rates continued to decrease. A limitation of this study is that the observed differences in prevalence may be due to changes in the study population over time, rather than true differences in prevalence. Longitudinal studies, such as the one conducted in this study, are more appropriate for investigating changes in prevalence.

Given the long-term impact of smoking behavior on the future burden of disease, knowledge of smoking behavior changes during the COVID-19 pandemic among adults is important for tobacco policies and preventive health efforts. In this context, our aim was to investigate smoking prevalence from 2009 to 2021 focusing on differences between educational level. Furthermore, we aimed to study the association between changes in smoking behavior from 2018 to 2021 and educational attainment.

## Methods

### Setting and study population

This study is based on data from 1785 participants of the French TEMPO cohort. TEMPO is a longitudinal follow-up study that aims to better understand which factors are associated with mental health and substance use, including cigarette smoking patterns. The cohort was set up in 2009 among young adults (22–35 years) who had previously taken part in a study on children’s psychological problems and access to mental health care between 1991 and 1999 [29]. TEMPO participants were followed via self-completed questionnaires in 2009, 2011, 2015, and 2018 [29]. Between March 2020 and April 2021, TEMPO cohort participants completed nine questionnaires aiming to better understand their experience of the COVID-19 pandemic. The first eight questionnaires were sent exclusively by email, and the ninth questionnaire was sent by either email or post. For further detail on the dates of assessment and number of participants, see Supplementary Figure S1.

### Measures

Participants’ self-reported smoking tobacco status was measured in 2009, 2011, 2015, 2018, 2020 and 2021 (daily, occasional, former smoker or non-smoker) and the average number of cigarettes smoked per day was recorded. The measure of tobacco use for 2020 included participants’ smoking status the first time they answered a COVID-19 questionnaire between March and July 2020 (during the first 8 waves of data collection), and the 2021 measure included data collected in wave 9 (collected from December 2020 to May 2021). All participants who reported smoking on average ≥ 1 cigarette per day were considered to be regular smokers [30].

Interpersonal change in cigarette smoking between 2018 (the last available measure before pandemic) and 2021 was measured using participants’ smoking status and if smoking, the number of cigarettes smoked in 2018 and in the latest available measurement of smoking status from the TEMPO COVID-19 questionnaire (from here the 2021 measurement) (for the majority from wave 9 (n = 535), wave 1–7 (n = 147) and wave 8 (n = 34)). We compared the individual change from 2018 to 2021 and categorized this into four groups (non-smoker, stable smoker, started or increased smoking, stopped or quit smoking). A 25% or more change in the number of cigarettes smoked daily was considered as a change in behavior, whether increasing or decreasing, to ensure that not only a small change e.g. 1 cigarette per day, would be considered as a change. Sensitivity analyses were performed with 10% and 50% as cut-off.

*Educational level* was accessed at all follow-ups. Educational level was dichotomized into low (high school level or less (Bac + 2 or lower)) vs. high (higher than high school (Bac + 3 or higher)). Other sociodemographic characteristics included participants’: *sex*, *age, symptoms of anxiety/depression (*using items from the Anxious/Depressed syndrome scale based on the Adult Self Report (ASR)-Achenbach System [31]) ) (yes, no), *household income in 2020* (≤ 2.500 €, ≥ 2.501 €), *working situation in 2020* (employed and normal work situation or working from home vs. unemployed, forced leave, loss of job, or sick leave) and whether or not they were living with a partner (yes, no),

### Statistical analysis

The prevalence of smoking was estimated at each time point from 2009 to 2021, stratified based on participants’ level of education (low vs. high) and weighted for non-response probability. The difference in smoking prevalence across educational level at each time point was tested using chi-square. T-tests were used to compare the difference of the prevalence of daily smoking between the two educational level groups in 2020 and 2021. To study the association between interpersonal changes in tobacco use from 2018 to 2021 and educational level, we used multinomial logistic regression with the outcome categories (1) stable smoker (reference) (2) increased or initiated smoking or (3) decreased or had quit smoking. We examined factors associated with changes in smoking behavior, and adjusted for those associated with a change by a significance level p < 0.2, including sex, age, living in a couple and symptoms of anxiety/depression. Furthermore, we examined the mean of number of cigarettes, and the absolute and percentage change from 2018 to 2021, in the two educational level groups. To account for differential attritition, non-response weights were calculated using Inverse Probability Weighting (IPW) [32, 33] including socio-demographic factors (including sex, education level, living in a couple, symptoms of anxiety or depression and alcohol use from 2009 onwards), and subsequently applied to all further analyses. For all statistical tests, a significance level p < 0.05 was used. Besides sensitivity analyses on the interpersonal change in cigarette smoking using a 10% and 50% cut-off in change in number of cigarettes,prevalence estimates were performed using a different cut-off for educational attainment (less than high school (baccalaureate or less) vs. highschool or more (Bac + 2)). Analyses were performed using SAS Software, version 9.4 (SAS Insitute, North Carolina, US).

## Results

In total, 1785 participants were included in analyses, with respectively: 1036 participants in 2009, 1211 in 2011, 753 in 2015, 837 in 2018, 696 in 2020 and 603 in 2021. Participants with missing information on smoking status at all time points (n = 18) or missing information on the covariates used to calculate the non-response weight (n = 52) were excluded. To study the interpersonal change from 2018 to 2021, 716 participants were included, of which 148 were smokers and thus included in the multinomial logistic regression (see **Supplementary Figure S2** for STROBE diagram). The study population were mostly female (62%), averaged 40 years old, and had a higher educational level (72% completed high school or above) than the general French population [29]. Table 1 shows the sociodemographic characteristics of the 716 participants included in analyses on the interpersonal change from 2018 to 2021, and shows that participants with lower level education were more likely to be stable smokers during the pandemic, whilst participants from the high educational level group were more commonly non-smokers. Furthermore, lower educated participants were more likely to be unemployed, or taking either forced leave, sick leave and maternal/paternal leave during the pandemic.

**Table 1:** Sociodemographic characteristics of the study population who answered in 2018 and to TEMPO COVID-19 (n = 716)

			Highest educational level*		
		Total n = 716	Bac + 3 or more n = 515	Bac + 2 or less n = 201	Chi^2^ p-value
Change in smoking situation	Non-smokers	568 (76.4%)	78.2% [74.5;82.0]	72.5% [65.8;79.1]	*0.0487*
	Stable smokers	33 (5.8%)	4.0% [2.1;5.9]	9.7% [5.1;14.3]	
	Initiated/Increased smoking	63 (9.8%)	9.4% [6.7;12.1]	10.5% [5.9;15.1]	
	Quit/Decreased smoking	52 (8.0%)	8.3% [5.8;10.9]	7.4% [3.6;11.2]	
Sex	Male	249 (37.7%)	37.1% [32.8;41.4]	38.9% [31.8;46.0]	0.6688
	Female	467 (62.3%)	62.9% [58.6;67.2]	61.1% [54.0;68.2]	
Age (years)	Mean (SD)	40.0 (3.62)	39.7[39.4;40.0]	40.5[40.0;41.0]	*0.0086*
Living in a couple (m*=5)	Yes	565 (78.3%)	78.3% [74.6;82.0]	78.4% [72.5;84.4]	0.9716
	No	146 (21.6%)	21.7% [18.0;25.4]	21.6% [15.6;27.5]	
Symptoms of anxiety/depression in 2020	No	585 (80.6%)	82.0% [78.6;85.5]	77.3% [71.2;83.5]	*0.1702*
	Yes	131 (19.4%)	17.9% [14.5;21.4]	22.7% [16.5;28.8]	
Household income in 2021 (m*=16)	≤ 2.500 €	129 (20.0%)	13.0% [9.7;16.0]	35.4% [28.4;42.3]	*< 0.0001*
	≥ 2.501 €	571 (80.0%)	87.0% [84.0;90.0]	64.6% [57.7;71.6]	
Working situation in 2020 (m*= 31)	Work normally or from home	497 (71.4%)	78.6 [74.8;82.3]	56.2 [49.0;63.4]	*< 0.0001*
	Unemployment, forced leave, loss of job, sick leave, maternal/paternal leave	188 (28.6%)	21.4 [17.7;25.2]	43.8 [36.6;51.0]	

*m for number of missing values

Prevalence are weighted for non-response

**Fig. 1:**
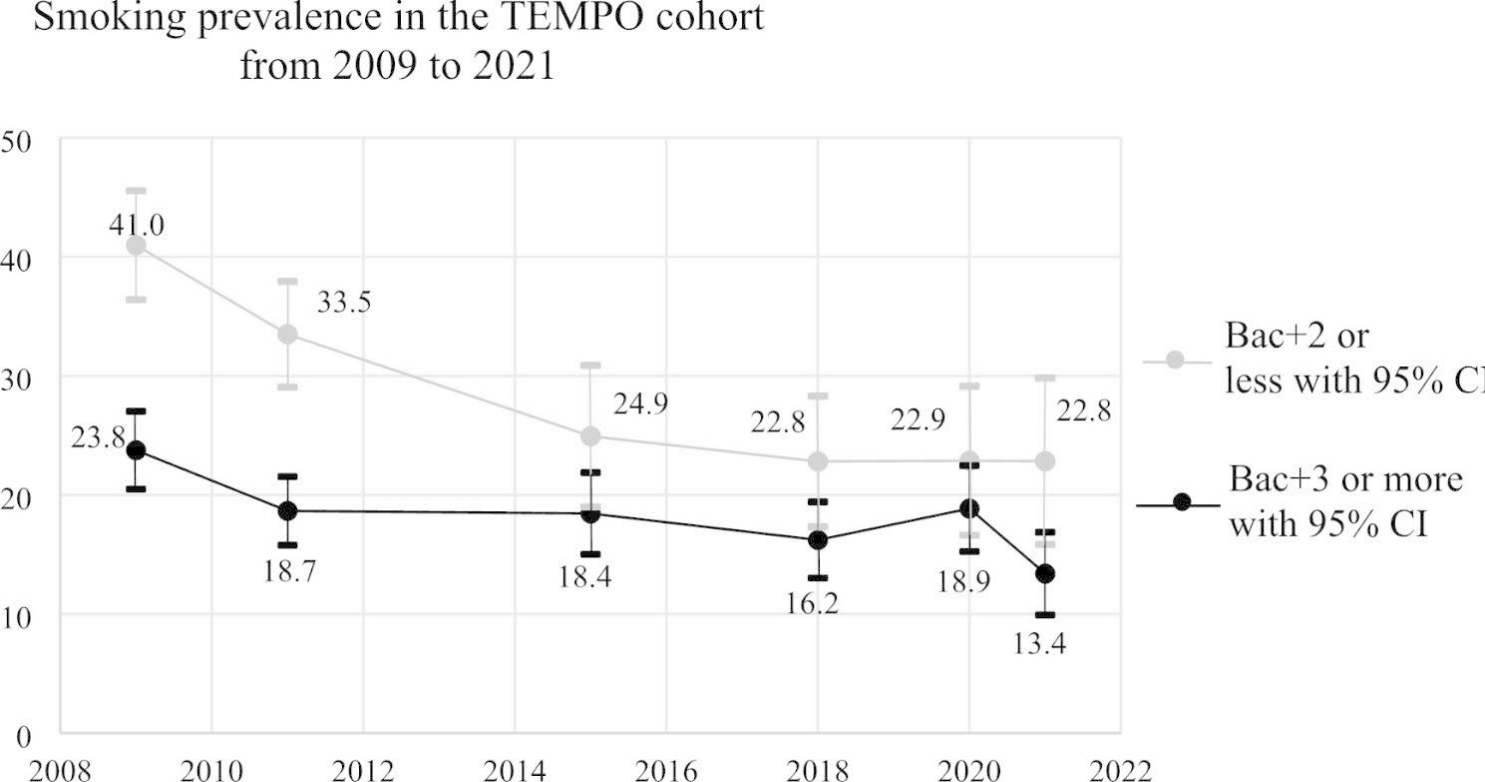
Smoking prevalence stratified by educational level (Bac + 3 or higher vs. Bac + 2 or less)

In Fig. 1, the smoking prevalence rates between 2009 and 2021 for individuals with low and high educational attainment are depicted. Except for the year 2015 and 2020, the prevalence of daily smoking was consistently higher among those with low educational attainment compared to those with high educational attainment across all time points (chi^2^ p-values; 2009 = < 0.0001, 2011 = < 0.0001, 2015 = 0.0523, 2018 = 0.0322, 2020 = 0.2597 and 2021 = 0.0093). The smoking prevalence estimates for the lower educational group were stable from 2018 to 2021, whereas the prevalence changed more among participants in the higher educated group. Thus, the gap in smoking prevalence between the two groups increased from 2020 to 2021, going from a difference of 4.0% points to 9.4% points (p < 0.001).

Besides showing an increased prevalence of smoking, participants with lower educational attainment also smoked more cigarettes per day in 2021 than the high educational level group (12.6 vs. 7.90 cigarettes per day, respectively (p = 0.053)).

Table 2 shows results from the unadjusted and adjusted multinomal logistic regression. We found that, between 2018 and 2021, smokers with high educational attainment had higher odds of decreasing their cigarette consumption, as opposed to staying stable, when compared to the less educated population (aOR = 2.72 [1.26;5.89]). Thus, people with a higher level of educational showed an increased risk of changing their consumption during the COVID-19 pandemic, compared to people with lower educational level. Furthermore, age was found to be negatively associated with increasing smoking (aOR = 0.87 [0.78;0.97]), meaning that older participants showed lower odds of increased smoking behaviour (as opposed to staying stable) when compared to younger participants.

**Table 2:** Multinomial logistic regression model. Unadjusted and adjusted odds ratio (OR) of increased smoking or decreased smoking between 2018 and 2021 vs. being a stable smoker (n = 148), with 95% confidence interval (CI)

		Unadjusted				Adjusted model			
Educational level		Increased vs. stable smoking OR [95% CI]	p-value	Decreased vs. stable smoking OR [95% CI]	p-value	Increased vs. stable smoking OR [95% CI]	p-value	Decreased vs. stable smoking OR [95% CI]	p-value
	≥ Bac + 3	2.18 [1.06; 4.49]	0.0346	2.75 [1.28; 5.93]	0.0097	1.94 [0.92;4.09]	0.0822	2.72 [1.26;5.89]	0.0108
	≤ Bac + 2	Reference		Reference		Reference		Reference	

Model is IPW for non-response, and adjusted for: sex, age, living in couple, and anxiety/depression symptoms

In the sensitivity analysis, in which we used a 10% and 50% cut-off for interpersonal change, respectively, we found similar estimates with a stronger association when using a 10% cut-off and weaker and statistically non-significant association using the 50% cut-off (Table S1 and S2). In the sensitivity analysis using a lower cut-off for educational level, we found similar trends in smoking prevalences, with a higher prevalence among those less educated and a larger difference between the two groups. We did not identify any significant difference in the absolute (p = 0.4358) or percentage (p = 0.2346) changes of daily cigarettes smoked from 2018 to 2021 between the two educational level groups.

## Discussion

### Summary of findings

Using data from a longitudinal cohort study that included pre-pandemic data as well as data collected during the COVID-19 pandemic, we found that, the prevalence of smokers was higher among people with low educational attainment compared to those with high. The gap between smoking prevalence across educational groups increased significantly between 2020 and 2021; The prevalence for those in the lower education group remaining stable from 2018 to 2021 whereas that of people with high educational level decreased. To our knowledge, this is the first study to examine longitudinal changes in smoking prevalence in the context of the COVID-19 pandemic, taking into account individuals’ educational attainment. Indeed, smokers with high educational attainment were more than twice as likely to decrease their smoking behavior compared to those with lower educational attainment.

### Study limitations and strengths

The interpretation of our results needs to be considered in light of several possible limitations. First, the size and composition of the study sample, which was primarily female, highly educated, and with a higher household income than the general French population [34], limit the generalizability of our results. However, the use of IPW to account for non-response is likely to have decreased the risk of selection bias. While the sample gives the study enough power to compare two education groups, the prevalence of smoking among persons belonging to the low education group is likely to be higher in the general population, as our cohort is not representative due to the participant selection [29]. Second, as no data was collected between 2018 and the onset of the COVID-19 pandemic, any changes during this period were not accounted for. However, the prevalence of tobacco smoking in this age group typically remains relatively stable under normal circumstances [35]. Due to the limited number of smokers that participated both in 2018 and after the onset of the COVID-19 pandemic (n = 148), we were not able to include measures for both 2020 and 2021 regarding the interpersonal change analysis. It would have been interesting to focus on 2020 to 2021 in terms of the timing of the increase and decrease in smoking behaviors in the two educational level groups, since our results indicates that the timing of data collection after onset of the pandemic has an impact on what is observed.

Moreover, our study evaluates only cigarette smoking and not tobacco smoking, which could have resulted in an underestimation of the actual level of tobacco use. Nevertheless, cigarettes (either pre-rolled or roll-your-own tobacco) are the most frequently used tobacco product in France, as in other countries [36], so the underestimation of use is likely to be minor. A broader assessment of the types of tobacco products that can be used (e.g. cigars, pipes, e-cigarettes), would have probably yielded larger estimates of socioeconomic inequalities in this area, as people with lower educational attainment are more likely to use non-cigarette tobacco products [25, 37, 38]. Despite these limitations, our study has several stengths which need to be highlighted. First, we used longitudinal data from the French TEMPO cohort, a community sample of young and middle-aged participants with information from 2009 to 2021, thereby limiting the recall bias observed in cross-sectional studies. TEMPO COVID-19 questionnaires were sent out 9 times during 2020 and 2021, enabling the possibility to account for multiple changes with regard to the COVID-19 pandemic, from both the beginning of the pandemic to one year after its onset.

### Interpretation

Our findings must be seen in the context of existing studies on smoking during the COVID-19 pandemic, where mixed results have been reported. Indeed, the meta-analysis from Sarich et al. (2022) [12] highlighted the divergent results from previous studies aiming to study changes in tobacco smoking before and during the COVID-19 pandemic. As highlighted in the meta analysis, few studies have looked at changes by educational level. A nationally representative, repeated cross-sectional study in the French general population [9], found that the difference in smoking prevalence between the lower and higher educational attainment groups did increase from 2019 to 2020. This was due to an increase among people with no diploma or a high school diploma, whereas the smoking prevalence remained stable among both higher and lower educated people. However, their results also indicated that the decline in smoking levels observed the last few years was halted by the COVID-19 pandemic among persons with lower educational attainment, which reflects that socioeconomic inequalities with regard to smoking increased between 2020 and 2021. As including anxiety/depression in the multinomial logistic regression model did not impact the odds-ratio of the educational level on smoking behavior, the association could be explained by other factors. Different factors may contribute to the explanation of the changes of the results. People with shorter educational attainment were found to be more likely to lose their job and to experience decrease in household incomes, which both are found to be associated with tobacco smoking. This can contribute to the explanation, that people with lower educational attainment were less likely to quit smoking from 2018 to 2020. On the other side, financial difficulties are found to be associated with higher quitting rates of smoking, and as financial difficulties are found to have affected those from low SEP the most during the pandemic. Unemployment has been found to be associated with greater risk for increase in tobacco use [39], which is in line with our findings and can be explained by individuals who were unemployed faced greater stress as a result of the pandemic.

Another study reported an increase in tobacco consumption in the first two weeks of lockdown in France [22], particularly among individuals’ aged 18–34 years, those with a high level of education, and those experiencing symptoms of anxiety. Importantly, this study was based on a self-evaluation of changes in smoking behavior, including questions about persons own perception of changes in smoking behavior, thus potentially not matching actual levels of tobacco consumption [40]. The divergent results according to changes in smoking behavior may also result from the timing of the data colletions, as we observed that the prevalence of smoking increased among those higher educated, but then decreased over time. This otherwise decline was not observed among those less educated, where a stable prevalence was observed from 2018 to 2021. The preventive measures introduced to limit the outbreak of COVID-19 were stressful, and the economic and financial impact of the pandemic has been found to be greatest among persons from low SEP [41]. Similarly, levels of anxiety and depression have been found to be highest among individuals with lower household income [26, 42, 43], which both associate with higher levels of smoking [7]. Before the pandemic, socioeconomically disadvantaged smokers were less likely to quit smoking, and successful attempts to quit consisted of a higher number of attempts [44, 45]. In terms of smoking cessation, life stress has been shown to impede attempts to quit smoking, in which smoking is seen to provide comfort and help cope with the stress [45]. The stress of the COVID-19 pandemic may have led to an increased exposure to both the risk factors of cigarette smoking, as well as hindrances towards quitting smoking, especially among individuals from low SEP. This group were both subjected to a higher risk of income loss, if their working hours were cud or loss of job, and also infection if they continued to work during the prevaccine period of COVID-19 [46]. Results from previous disease epidemics also showed that healthcare and social care professionals were at greater risk of developing short- and long-term mental health problems [47]. These societal factors contribute to the explanation of our results, where an increase was observed among smokers with higher educational attainment from 2018 to 2020, but 9–14 months after the onset on lockdown they then decreased their rate of smoking again. However, this trend was not observed among those with lower educational attainment, for which the smoking prevalence remained stable. Furthermore, lower educated smokers smoked on average more cigarettes than smokers with higher education.

### Implications

Examining how educational levels may influence tobacco smoking in context of the COVID-19 pandemic is a critical issue to address, since identifying groups who are most at risk of changing their smoking behavior can inform public health policies. It is important to identify both vulnerable groups and the factors associated with smoking prevalence, since the harms of tobacco use varies according to SEP. Not only is the incidence of disease, disability, and premature death higher among people with low SEP, but disadvantaged smokers are also at increased risk of dying from smoking, with tobacco responsible for about half of the socioeconomic-related differences in death rates among persons aged 35 to 69 [45]. Furthermore, research also indicates that smoking has an impact on the long-term labour outcome, indicating that smokers have lower income than non-smokers [48], which underlines a two-way causal effect between smoking and SEP, and the importance of preventive measures on tobacco smoking. Specific social and health policies are needed to decrease the socioeconomic gap in smoking behavior, particularly in the context of an epidemic crisis. Smoking cessation interventions, including individual-level approaches, have been shown to be effective for persons from both low and high SEP [49]. However, persons from more disadvantaged groups are still less likely to successfully quit smoking, even though they are just as likely to try to stop [45]. In France, smoking cessation advice from health care providers is well known to be associated with increased quitting [50], but during periods of the COVID-19 pandemic, where access to preventive and general medicine were limited, this path to smoking cessation was also limited.

## Conclusion

This study provides important evidence indicating an increased gap between smoking prevalence between educational attainment groups, during a pandemic period. These findings are important from a public health perspective, as changes in the populations smoking patterns following the COVID-19 pandemic may become habitual and persist after lockdowns have ceased. However, a longitudinal investigation is needed to provide more information about the impact of changes over time, especially to observe whether smoking behaviours during the COVID-19 pandemic will persist beyond the pandemic or will be only transitory. It is important to consider that “even small deviations from unstable steady state consumption can lead to large cumulative rises over time in addictive consumption or to rapid falls in consumption to abstention” [51]. Given that tobacco smoking is a habit-forming behavior, changes during the pandemic, including a potential rise in smoking, are predicted to persist since quitting smoking has low success rates, particularly among individuals with lower SEP [45].

## Electronic supplementary material

Below is the link to the electronic supplementary material.

Supplementary Material 1

## Data Availability

Due to the personal questions asked in this study, research participants were guaranteed that all raw data will remain confidential. On reasonable request including standards for General Data Protection Regulation data can be accessed, please send an email to cohort.tempo@inserm.fr. Anonymized data can only be shared after explicit approval of the French national committee for data protection for approval (Commission Nationale de l’Informatique et des Libertés, CNIL).
