# Supplementary material for: Are we equally at risk of changing smoking behavior during a public health crisis? Impact of educational level on smoking from the TEMPO cohort
Source: BMC Public Health. 2023 May 30;23:1016. doi: 10.1186/s12889-023-15799-1 (PMC10227809; doi:10.1186/s12889-023-15799-1)
Supplement: Supplementary file 1 — Supplementary Material 1 [file 12889_2023_15799_MOESM1_ESM.docx]

**Figure S1: Flow diagram for the TEMPO cohort and the TEMPO COVID-19 cohort.**


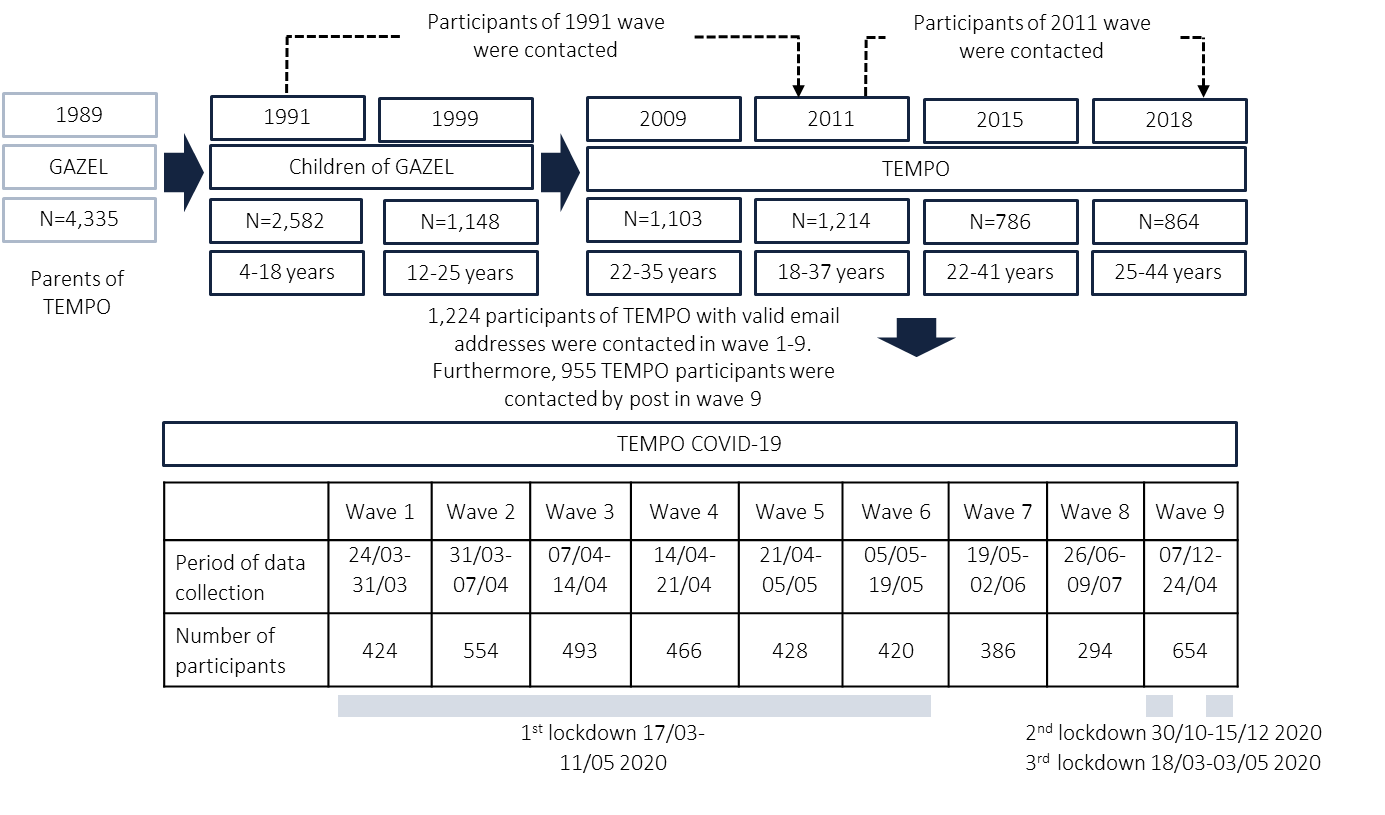


**Figure S2: STROBE diagram. Study population for respectively prevalence estimates, interpersonal change between 2018-2020 and 2020-2021.**


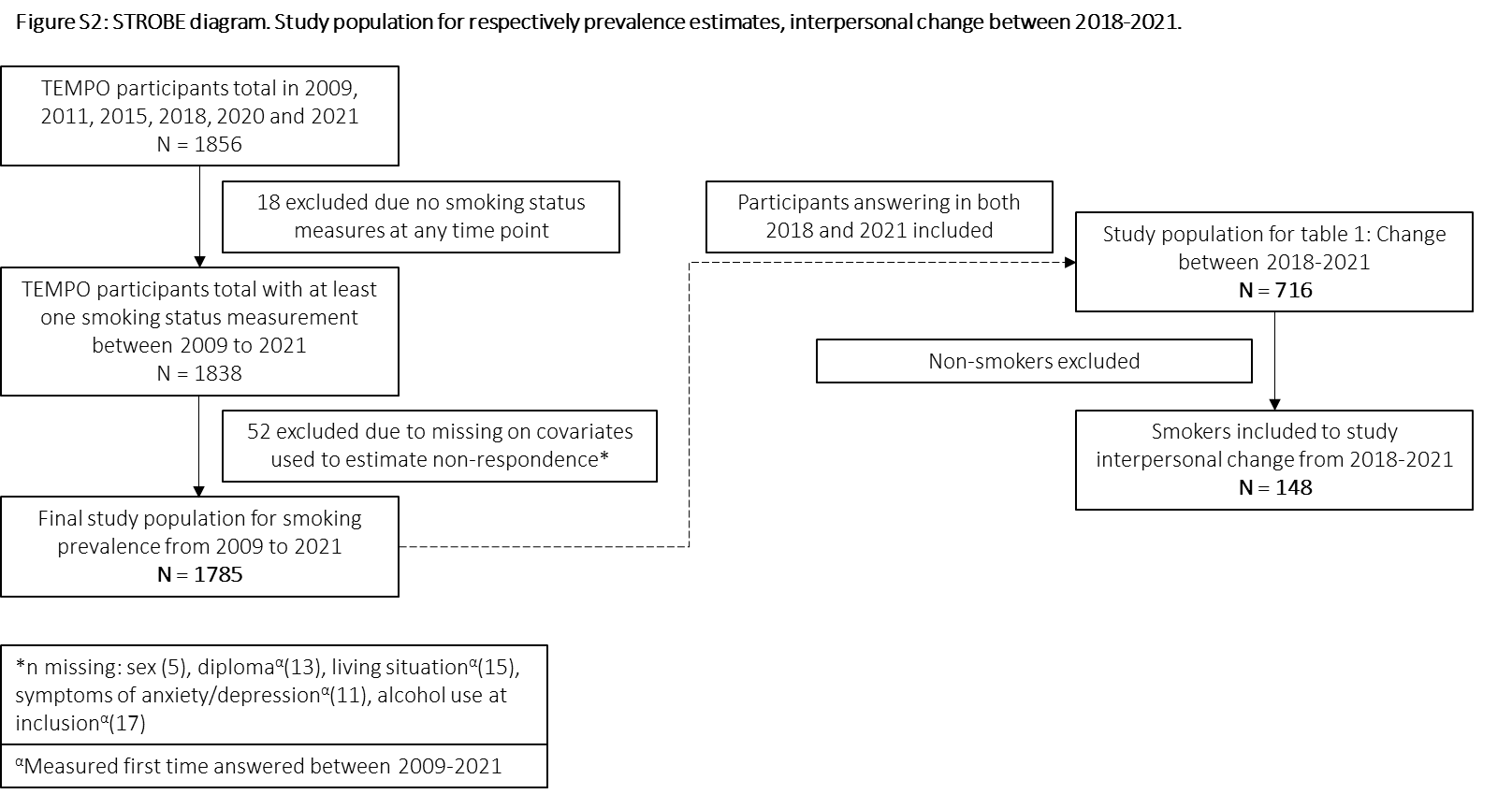


**Table S1: Multinomial logistic regression model using a 10% change in cigarettes smoked as cut-off. Unadjusted and adjusted odds ratio (OR) of increased smoking or decreased smoking between 2018 and 2021 vs. being a stable smoker (n=148), with 95% confidence interval (CI)**

|  | | Unadjusted | | | | Adjusted model | | | |
| --- | --- | --- | --- | --- | --- | --- | --- | --- | --- |
| Educational level | | Increased vs. stable smoking  OR [95% CI] | p-value | Decreased vs. stable smoking  OR [95% CI] | p-value | Increased vs. stable smoking  OR [95% CI] | p-value | Decreased vs. stable smoking  OR [95% CI] | p-value |
|  | ≥ Bac+3 | 2.56 [1.15; 5.69] | 0.0217 | 3.30 [1.43; 7.63] | 0.0053 | 2.28 [1.00;5.20] | 0.0492 | 3.27 [1.41;7.58] | 0.0058 |
|  | ≤ Bac+2 | Reference |  | Reference |  | Reference |  | Reference |  |

**Table S2: Multinomial logistic regression model using a 50% change in cigarettes smoked as cut-off. Unadjusted and adjusted odds ratio (OR) of increased smoking or decreased smoking between 2018 and 2021 vs. being a stable smoker (n=148), with 95% confidence interval (CI)**

|  | | Unadjusted | | | | Adjusted model | | | |
| --- | --- | --- | --- | --- | --- | --- | --- | --- | --- |
| Educational level | | Increased vs. stable smoking  OR [95% CI] | p-value | Decreased vs. stable smoking  OR [95% CI] | p-value | Increased vs. stable smoking  OR [95% CI] | p-value | Decreased vs. stable smoking  OR [95% CI] | p-value |
|  | ≥ Bac+3 | 1.24 [0.65; 2.38] | 0.5167 | 1.56 [0.72; 3.36] | 0.2577 | 1.09 [0.56;2.14] | 0.7978 | 1.58 [0.73;3.42] | 0.2515 |
|  | ≤ Bac+2 | Reference |  | Reference |  | Reference |  | Reference |  |
